# Defensive Strategies and Handling Paths in Intimate Relationship Conflicts: A Dynamic Game Model From the Perspective of Emotional Regulation

**DOI:** 10.1002/pchj.70103

**Published:** 2026-07-18

**Authors:** Yiwen Liu

**Affiliations:** ^1^ The Education University of Hong Kong Hong Kong China

**Keywords:** conflict, defense strategy, dynamic game model, emotional regulation, intimate relationship, processing path

## Abstract

This study develops a dynamic game model of intimate relationship conflicts that incorporates emotion regulation mechanisms, translating cognitive reappraisal and expressive suppression into strategic parameters. The model simulates the dynamic evolution of defensive strategies and intervention paths, capturing the continuous interplay between emotional states and conflict behavior choices in realistic relational contexts. Using longitudinal tracking data from 320 couples and agent‐based simulations, we validate the core mechanisms and demonstrate that flexibility in emotion regulation significantly reduces conflict intensity and shortens conflict cycles. When one partner's emotion regulation capacity is limited, power asymmetry intensifies defensive rigidity, highlighting the combined influence of neural coordination, relational fluidity, and micro‐level cultural practices on conflict dynamics. By operationalizing emotion regulation as a computable variable within a formalized game‐theoretic framework, this research bridges theoretical constructs and observable behavior, revealing how neural synchrony and relational context shape the selection and effectiveness of defensive strategies. The findings offer both theoretical and practical contributions: theoretically, the study provides a more integrated understanding of intimate relationship conflicts, emphasizing the joint roles of emotional, relational, and cultural factors in conflict evolution; practically, the model equips clinicians and family intervention practitioners with a structured, quantifiable tool for diagnosing conflict patterns and designing evidence‐based strategies to reduce destructive behaviors and foster cooperative problem‐solving. Overall, this study presents a concise yet comprehensive framework that advances both the conceptualization and empirical analysis of intimate relationship conflicts, offering actionable insights for intervention while highlighting the importance of integrating emotional, neural, and cultural dimensions in research and practice.

## 1 Introduction

In modern urban households, conflicts within intimate relationships have become a significant factor affecting social stability. Data indicate that 73% of marital conflicts stem from an imbalance in power distribution; 17% of escalating conflicts are directly related to changes in relationship fluidity, manifested as an increased willingness among individuals to change partners, leading to a decrease in commitment (Zhu et al. 2025). Over half of conflict incidents are accompanied by ineffective emotional regulation, triggering a vicious cycle ranging from conceptual disagreements to emotional alienation. These phenomena not only exacerbate family dysfunction but also significantly drive up divorce rates and social governance costs. It is urgent to explore the mechanisms of conflict generation and resolution paths from the micro‐interaction level.

Existing research on the interpretation of intimate relationship conflicts has primarily focused on static factor analysis, failing to fully reveal the dynamic and systematic nature of conflict evolution (Laicher et al. 2025; Rombouts et al. 2025). Although emotion regulation theory elucidates the strategic differences between cognitive reappraisal and expressive suppression, it overlooks the crucial role of neurobehavioral coordination in interpersonal interactions. Game theory models emphasize the distribution of power resources, but neglect to integrate the deep shaping of cultural values on conflict management approaches. This theoretical fragmentation makes it difficult for existing frameworks to explain why the same strategy can have vastly different effects in different relational contexts, what mechanisms can simultaneously optimize short‐term conflict mitigation and long‐term relationship stability, and how to break through these limitations by constructing an integrated model that incorporates emotion regulation, neurobehavioral coordination, and cultural constraints.

The essence of conflict in intimate relationships is a three‐dimensional dynamic game involving emotional needs, power structures, and values. Defense strategies exhibit a hierarchical nature in this process: primary avoidance strategies can temporarily cool down high arousal states but may exacerbate power imbalances; advanced engagement strategies such as cognitive restructuring and event intervention, although promoting fundamental resolution, rely on the neural coordination efficiency and cultural consensus of both parties (Li, Huang, and Ding 2025; Li, Zhao, and Jeon 2025). Current research has not yet clarified the mechanism linking the choice of defense strategies to the equilibrium point of the game, nor has it specified how neural coordination thresholds regulate strategy effectiveness. This lack of integration renders conflict intervention programs lacking in precision and universality.

This study aims to reveal the dynamic game rules of intimate relationship conflicts, connecting defensive strategy selection, neural coordination mechanisms, and cultural context constraints through the perspective of emotional regulation. Although previous research has examined emotion regulation or power and value factors, most studies are limited to static analysis or single theoretical frameworks, lacking a systematic integration of emotion regulation, neural synchrony, and cultural norms in the dynamic evolution of conflicts. This study explores this gap by proposing a dynamic game model that includes emotion regulation. Based on empirical examination of neural, relational, and cultural factors, the results provide preliminary theoretical insights and a possible practical framework for conflict intervention. Specific objectives include: analyzing the interactive evolutionary path of three‐dimensional conflicts involving power, emotion, and perception; verifying the adaptive boundaries of defensive strategies under different relationship fluidity; and establishing the moderating effects of neural coordination and cultural factors. The research findings will provide a theoretical foundation for family conflict intervention, promote the development of a precise intervention path of “prevention‐mitigation‐restoration,” and help build a more resilient intimate relationship ecosystem.

It should be noted that the present study proposes an integrated dynamic‐game framework, whereas the empirical analysis focuses on three observable and testable pathways within that framework: the pathway from relational fluidity to goal selection and destructive coping, the boundary role of interbrain synchrony in emotion‐regulation strategy effectiveness, and the buffering effect of benevolence‐righteousness dissemination on conflict recurrence. In other words, the study does not attempt to exhaustively test the entire theoretical system within a single dataset; rather, it prioritizes the operational verification of its core mechanisms so as to strengthen the alignment between theoretical construction and empirical testing.

## 2 Literature Review

### 2.1 Three‐Dimensional Structure of Intimate Relationship Conflict

The essence of conflict in intimate relationships lies in the dynamic game of resource allocation and decision‐making dominance. In empirical research, this conflict structure of resource allocation and decision dominance can be operationalized through a contextualized resource‐allocation game, thereby translating the abstract notion of relationship conflict into observable behavioral choices. Research indicates that 73% of marital conflicts stem directly from an imbalance in power structures, which is primarily manifested in the unequal division of household chores, unequal economic dominance, and competition for the right to speak in major decisions (Bachfischer and Harris 2025). This power game has both explicit and implicit dimensions: the explicit level manifests as disputes over responsibility allocation and conflicts in behavioral control; the implicit level involves competition for emotional discourse power. Power imbalances trigger defensive confrontation mechanisms, leading conflicts to escalate from specific incidents to questioning the value of the relationship, ultimately evolving into persistent relationship tension. The threshold for resolving power games exists in 78% of balanced samples, confirming that reasonable power allocation is the cornerstone of conflict resolution (Guo et al. 2025; Tian and Zhang 2025).

**FIGURE 1:**
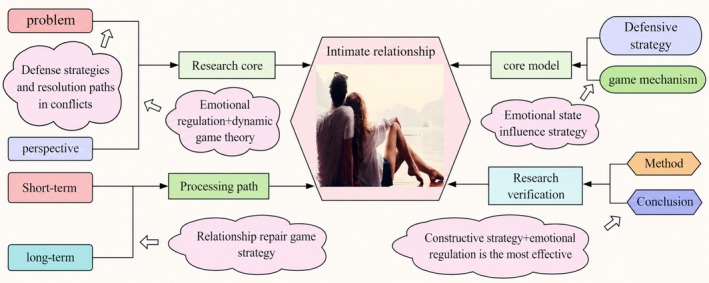
Article structure diagram.

The misalignment of emotional needs and divergences in values constitute the other two core dimensions of conflict (Li, Huang, and Ding 2025; Li, Zhao, and Jeon 2025). As shown in Figure 1, emotional coordination relies on bidirectional empathy and emotional regulation efficacy. Its neural basis is reflected in the significant improvement in emotional understanding efficiency when the synchronous activation level of specific brain regions exceeds the threshold of 0.6 (Nazzari et al. 2025). Conceptual conflicts are deeply rooted in cultural backgrounds and individual socialization differences. Among individuals over 35 years old, 61% of conflicts involve intergenerational parenting views or differences in family role cognition. The three‐dimensional structure does not exist in isolation. Power imbalances weaken the willingness to respond emotionally, differences in values exacerbate the justification of power struggles, and emotional fractures hinder the reconstruction of value consensus (Schrader et al. 2025). This interaction forms a “conflict spiral.” Only through a neurobehavioral coordination mechanism can the negative cycle be broken, and relationship repair be achieved in the simultaneous process of power redistribution, emotional reconnection, and conceptual reintegration.

### 2.2 Dual‐Path Adaptation of Defense Strategy

As a primary defense mechanism, the efficacy of avoidance strategies is highly dependent on the relational context and developmental stage. In early adolescent groups, 42% of conflict resolution benefits from temporary emotional withdrawal, which prevents conflict escalation by blocking high arousal states (Alsubai et al. 2025). The essence of this strategy lies in the protective inhibition of neural resources, based on the negative feedback regulation of the prefrontal‐limbic system. Its applicability has clear boundaries. In low‐fluidity relationships with power balance, avoidance can create emotional cooling space; however, in high‐fluidity or power imbalance relationships, avoidance is easily interpreted as emotional rejection, which in turn exacerbates relational alienation (Vogl et al. 2025). The time‐sensitive characteristics of primary defense require precise identification of the initiation window, and overuse will lead to conflict solidification.

**FIGURE 2:**
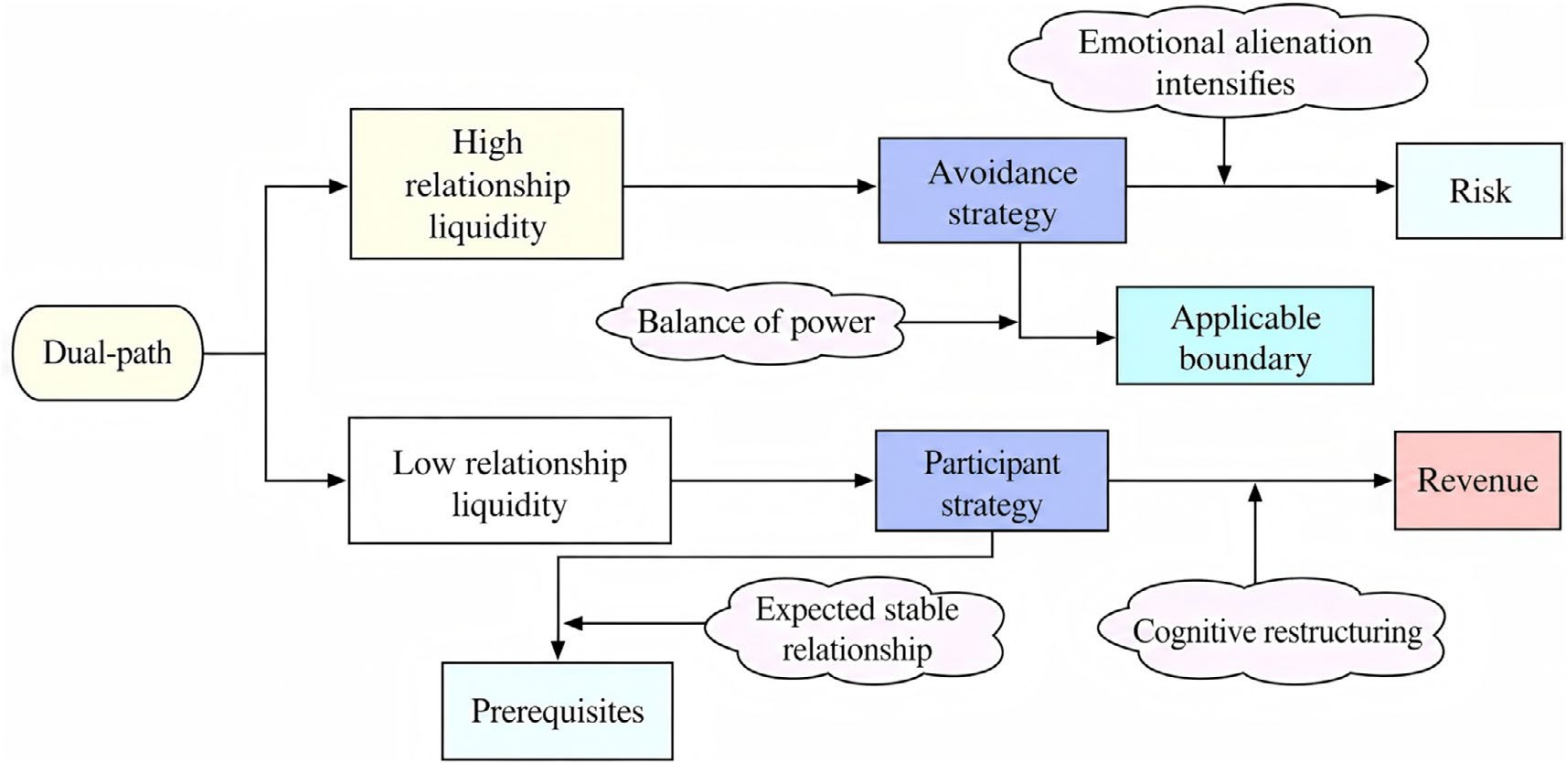
Defense dual‐path adaptation diagram.

As shown in Figure 2, the participatory strategy, as an advanced defensive pathway, relies on active coordination behaviors such as cognitive reconstruction and event intervention. Its efficacy is built on a dual foundation: at the neural level, it requires the brain synchronization intensity in the right temporoparietal junction to exceed a threshold of 0.6, ensuring mutual empathetic understanding and cognitive integration; at the social level, it requires relationship fluidity to be within a stable range, allowing strategies such as value evaluation and social comparison to have room for implementation (Scheffel and Gärtner 2025; Kilany and Mahfouz 2025). This pathway transforms adversarial game theory into collaborative problem‐solving by reconstructing the conflict meaning system. The dominance of the participatory strategy among late adolescents confirms the interaction between prefrontal cortex maturation and social cognitive development. The dual‐pathway adaptation model reveals that the selection of defensive strategies is essentially a dynamic allocation process of neural and social resources.

### 2.3 Core Mechanism of Dynamic Game

Previous research has examined conflict from emotion regulation, power game, and cultural norm perspectives, but these frameworks often exist independently without integration. This study systematically synthesizes these three perspectives, illustrating how emotion regulation strategies operate within power structures and cultural constraints, providing a coherent theoretical foundation for the subsequent dynamic game model. The game‐theoretic essence of conflict in intimate relationships manifests as resource competition between individual goals and relationship goals. Relationship fluidity, as a core moderating variable, affects goal priority allocation by altering expectations of relationship stability (Yan et al. 2025). In high‐fluidity environments, individuals tend to intensify investment in personal goals and direct emotional resources toward self‐development needs, leading to conflict coping strategies characterized by short‐term utilitarianism; low‐fluidity situations activate mechanisms for maintaining relationship goals, prompting individuals to suppress personal needs in exchange for relationship stability (Yu et al. 2025). This dynamic coupling reveals the deep logic of conflict evolution: fluidity levels determine the spatiotemporal horizon of game strategies, thereby shaping a continuous behavioral spectrum ranging from adversarial competition to collaborative adaptation. The goal selection mechanism under different fluidity levels constitutes the primary driver of differentiated conflict resolution paths.

**FIGURE 3:**
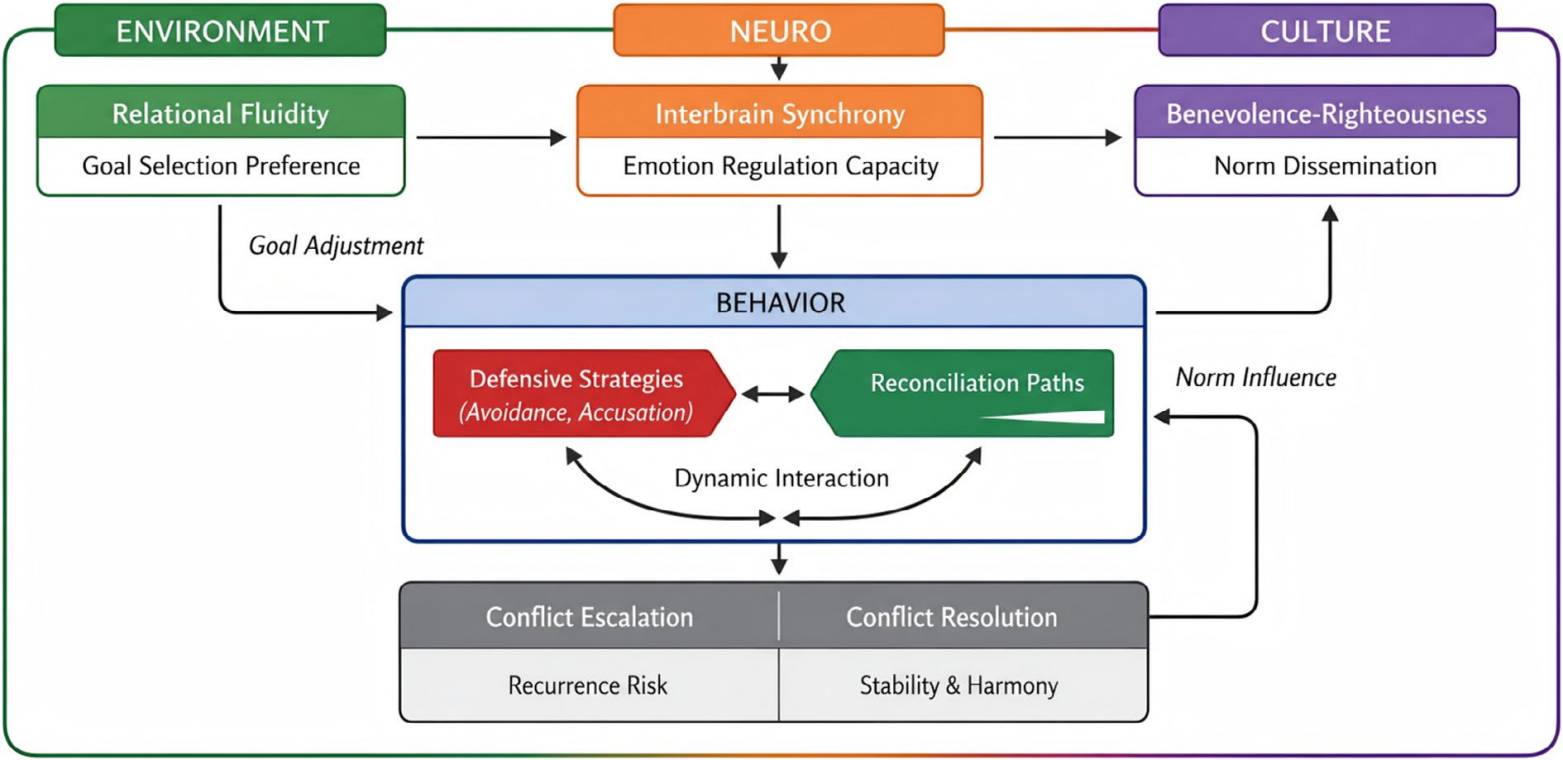
Integrated conceptual framework of the dynamic game mechanism.

To improve accessibility for readers from different disciplines, an integrated conceptual figure is added here to summarize the relationships among environmental cues, neural coordination, cultural norms, and behavioral outcomes. The figure captures the overall logic of the dynamic game mechanism: relational fluidity shapes goal selection, interbrain synchrony conditions emotion‐regulation efficiency, and benevolence‐righteousness dissemination structures interaction norms, with their joint effects reflected in conflict coping and subsequent outcomes (Figure 3).

This figure provides an overview of the integrated framework, and the following sections elaborate the environmental pathway, the neural threshold mechanism, and the cultural buffering effect in greater detail.

**FIGURE 4:**
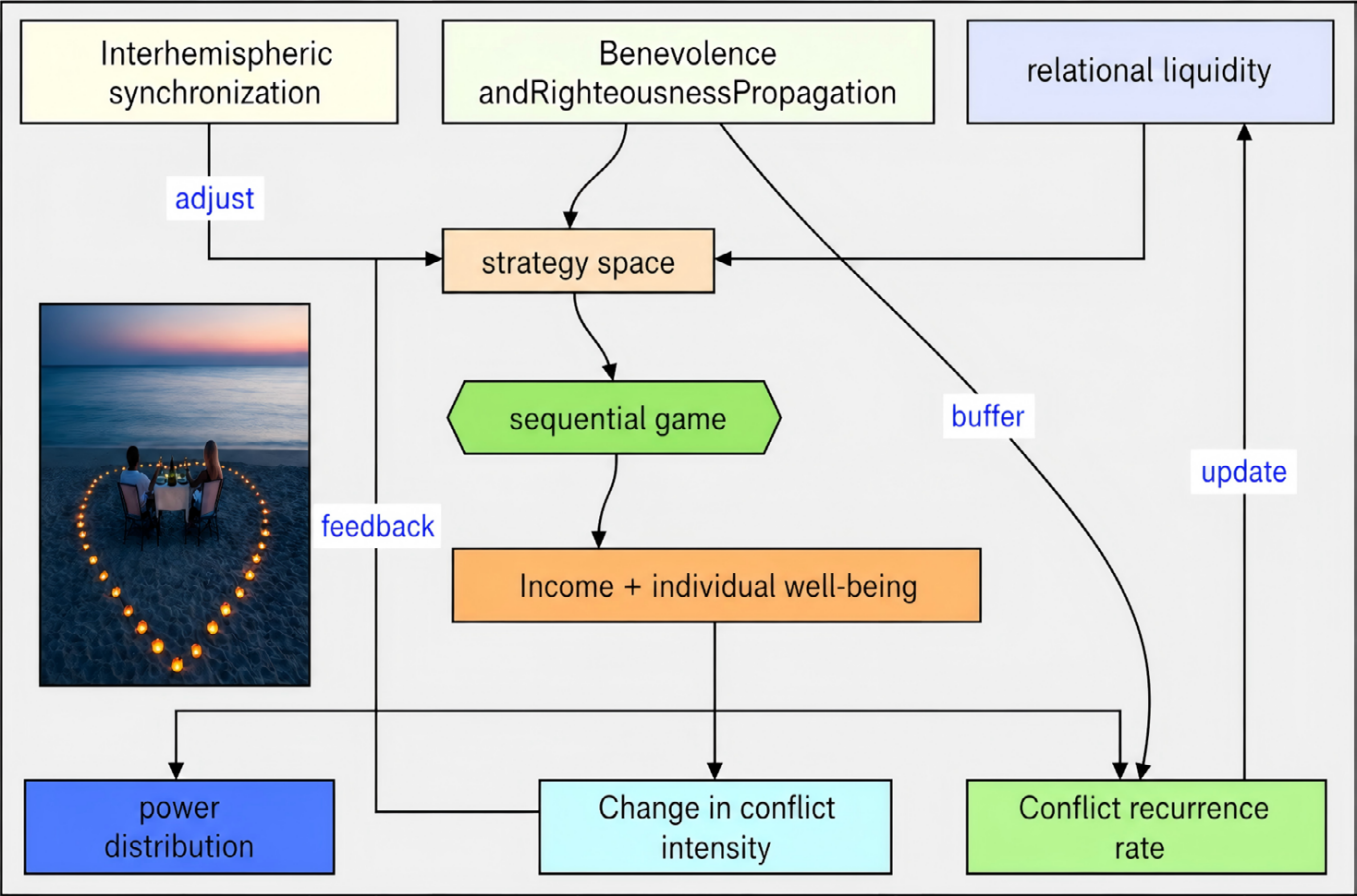
Core mechanism diagram.

The game process is simultaneously constrained by cultural norms and neural rigidity. As shown in Figure 4, the dissemination of benevolence and righteousness, as a cultural moderating variable, guides conflict games toward a value framework of shared responsibility through three behavioral paradigms: respect, gratitude, and support significantly reducing the risk of relationship breakdown. Its mechanism of action is reflected in the reconstruction of game rules: transforming zero‐sum competition into positive‐sum interaction, giving moral legitimacy to power redistribution. Neural coordination provides the physiological basis for game efficiency. When specific brain regions are simultaneously activated beyond a threshold level, the cognitive integration and emotional resonance abilities of both parties are significantly enhanced, providing neural resource support for the implementation of complex strategies (Saluja et al. 2025; Peng et al. 2025; Giovanni 2025). The interaction between cultural rules and neural efficiency jointly anchors the equilibrium boundaries of dynamic games, ensuring that conflict resolution is both socially adaptable and physiologically sustainable.

### 2.4 Research Hypothesis

To clarify the conceptual framework, this study summarizes the core relationships among variables. Relational fluidity, as the independent variable, influences goal selection preference, which in turn affects destructive coping. Neural synchrony moderates the effectiveness of cognitive reappraisal strategies. Meanwhile, benevolence‐righteousness dissemination, as a cultural variable, buffers the positive impact of relational fluidity on conflict recurrence. These relationships form the logical basis for the subsequent hypotheses and model analysis.

Based on a systematic study of individual resource allocation strategies from the perspective of relational fluidity theory, it has been found that high fluidity environments significantly strengthen individual goal orientation, prompting individuals to prioritize self‐satisfaction in intimate relationships and weakening relational investment, thereby leading to an escalation of defensive behaviors (Xu and Zhao 2025; Chen et al. 2025; Gao et al. 2025). This phenomenon reveals a chain reaction mechanism in which fluidity levels influence conflict coping behaviors through the mediating path of goal selection. Accordingly, this study proposes the core hypothesis H1: High relational fluidity situations will positively predict destructive coping tendencies, with its effect partially achieved by enhancing individual goal selection preferences. This hypothesis aims to verify the distal driving path and its internal mechanism of action of fluidity environments on conflict behaviors.

**FIGURE 5:**
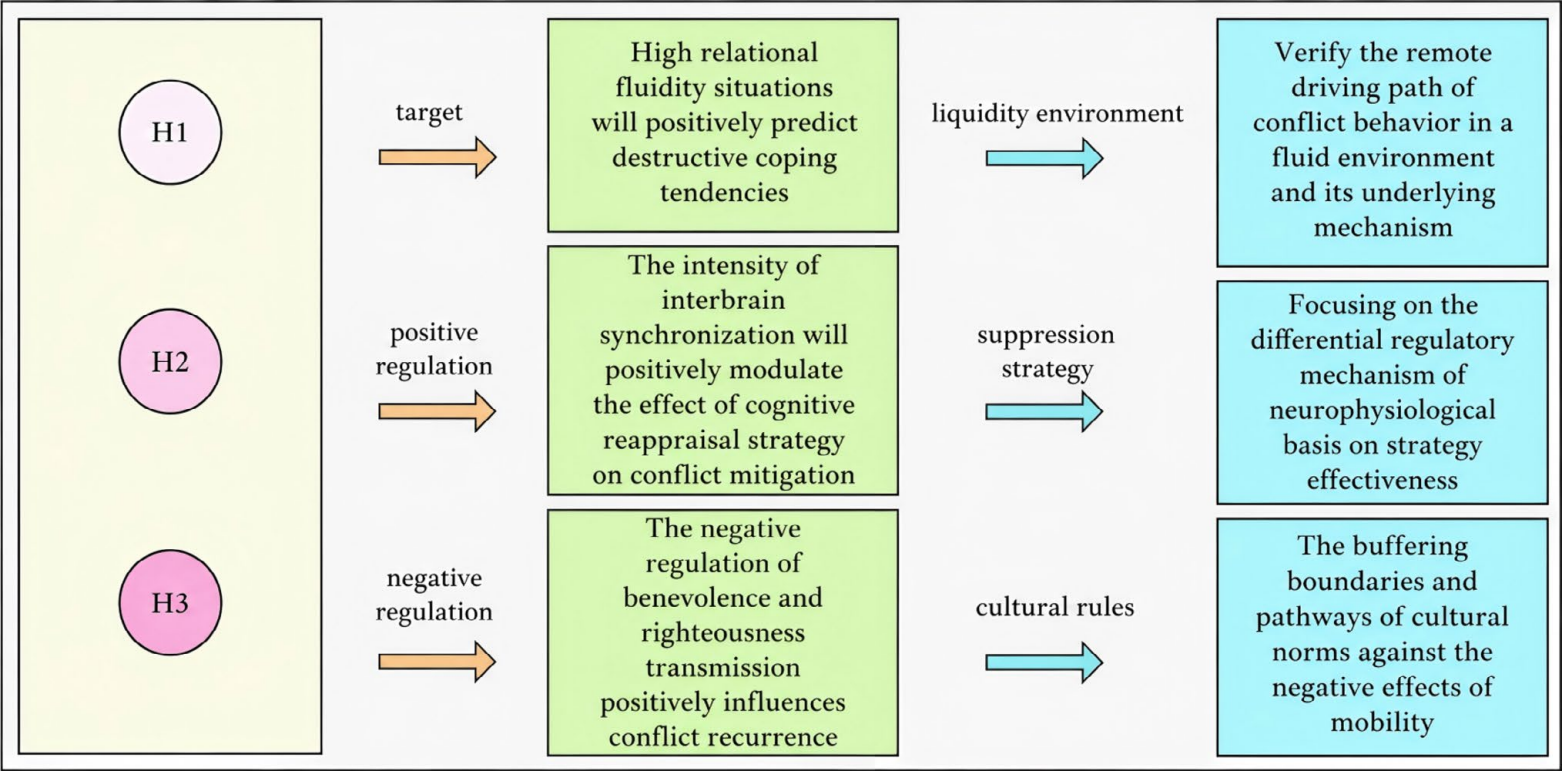
Research hypothesis diagram.

In‐depth exploration of interpersonal emotion regulation in the field of neurosocial science has shown that when interbrain synchrony reaches a specific threshold level, it can significantly optimize the cognitive processing efficiency of higher‐order emotion regulation strategies (Jing et al. 2025). As illustrated in Figure 5, this discovery reveals that the level of neural coordination has a clear efficacy boundary effect on complex strategies such as cognitive reappraisal. When the coordination intensity is insufficient, the implementation of strategies will be limited by the neural resource integration capacity. Based on the theoretical framework of neurobehavioral coupling, this study proposes the key hypothesis H2: The intensity of interbrain synchrony will positively modulate the effect of cognitive reappraisal strategy on conflict mitigation, while this moderating effect is not significant in the expressive suppression strategy. This hypothesis focuses on the differential regulatory mechanism of neurophysiological basis on strategy efficacy.

Cross‐cultural research on the interpretation of Eastern ethical paradigms has established the three‐dimensional behavioral characteristics of the benevolence and righteousness dissemination model. By reconstructing game rules, it transforms adversarial interactions into a collaborative framework of shared responsibility, effectively inhibiting the tendency for conflict recurrence (Fedorov et al. 2025; Yang et al. 2025). This theoretical finding emphasizes the corrective function of cultural norms on the evolutionary path of conflict, especially in providing a stable anchor for relationships in high‐mobility environments. Based on the regulatory patterns of cultural variables on conflict dynamics, this study proposes the moderating effect hypothesis H3: the level of benevolence and righteousness dissemination negatively moderates the positive impact of relational fluidity on conflict recurrence, with its protective effect materializing in the three‐dimensional behaviors of respect, gratitude, and support. This hypothesis aims to examine the buffering boundaries of cultural rules against the negative effects of mobility and their behavioral implementation pathways.

It should be noted that the “cultural context” examined in this study does not refer to macro‐level national cultural dimensions, but rather to a normative cultural mechanism enacted in intimate interaction. In the present research, this mechanism is operationalized through the three behavioral dimensions of benevolence‐righteousness dissemination, namely respect, gratitude, and support. Theoretically, it is closer to interaction‐level relational norms and value enactment than to macro‐cultural orientations such as individualism–collectivism or power distance; it is therefore positioned here as a micro‐level cultural regulatory mechanism.

## 3 Research Design

### 3.1 Mixed Methods Design

This study adopted a 2 × 2 between‐subjects experimental design, systematically manipulating the dual independent variables of relationship fluidity and emotional regulation strategies (Li and Zhao 2025). The total sample size was 200. Relationship fluidity was precisely manipulated through a situational simulation paradigm: the high fluidity group received social substitution cue stimulation, while the low fluidity group was reinforced with relationship stability cues. Emotional regulation strategies employed standardized training protocols, with the cognitive reappraisal group learning event meaning reconstruction techniques and the expressive suppression group training emotional behavior suppression abilities. The experiment set up a resource allocation game task, requiring participants to allocate 100 units of virtual funds between personal development resources and relationship investment resources, quantifying target selection preferences.

Specifically, after completing the scenario priming and emotion‐regulation training, participants read a standardized intimate‐relationship conflict vignette and were asked to allocate 100 units of virtual resources between “personal development investment” and “relationship repair investment.” The system recorded the allocation ratio, the initial choice direction, and the final adjustment result, and the difference between person‐oriented and relationship‐oriented allocation was used as a continuous indicator of goal‐selection preference. This paradigm maps onto the “relationship conflict” stated in the title because intimate relationship conflict is conceptualized here as a distributive game involving limited resources, emotional demands, and relational obligations, and the allocation bias directly reflects defensive versus cooperative tendencies in conflict situations. The experiment closely mirrors real‐life conflict situations through scenario‐based tasks, including watching standardized conflict videos and allocating virtual resources reflecting relational investment versus personal development preferences, thereby enhancing the ecological validity of the results.

Accordingly, the resource‐allocation game is not intended to replace relationship conflict itself but to serve as an operational tool for measuring goal orientation and strategic choice under conflict conditions.

At the empirical level, the integrated framework is decomposed into three measurable modules: resource‐allocation outcomes are used to capture goal‐selection preferences and their behavioral consequences, interbrain synchrony indices are used to represent neural coordination conditions, and behavioral coding of benevolence‐righteousness dissemination is used to capture the interactive enactment of cultural norms. Through this modular design, the environmental, neural, and cultural dimensions in the theoretical model are linked to observable variables and translated into a testable research design.

The sample demographics were as follows: all participants were in stable intimate relationships, with a mean age of 29.84 years (SD = 5.71); 54.00% were women and 46.00% were men. In terms of occupation, enterprise employees, public‐sector staff, and self‐employed participants accounted for 41.50%, 28.00%, and 30.50%, respectively, and monthly income was concentrated mainly in the middle range. No significant between‐group differences were found in these demographic characteristics, indicating basic comparability across conditions. These demographic variables are also reported as boundary‐condition information, helping to clarify the applicable scope of the model and its potential limits of generalizability across different socioeconomic backgrounds.

Neural activity recording utilized functional near‐infrared spectroscopy technology, focusing on the interbrain synchrony in the right temporoparietal junction area. During the synchronous scanning process by two people, the subjects jointly watched a standardized intimate conflict scenario video, followed by a 10‐min open‐ended discussion on conflict resolution (Ryan et al. 2025). The core indicator was the phase lock value of the blood oxygen‐dependent signal, which was calculated using sliding time window analysis, and the formula was defined as follows:

(1) $$\mathrm{PLV}=\left|\frac{1}{N}\sum_{t=1}^{N}e^{i\left[\varphi_{1}\left(t\right)-\varphi_{2}\left(t\right)\right]}\right|$$

where φ represents the instantaneous phase of the signal, N, which is the total number of time points. Simultaneous collection of skin conductance response and heart rate variability serves as physiological evidence (Fedorov et al. 2024).

The resource allocation game involves 20 rounds of decision‐making, with a mandatory allocation of 100 units of resources per round. The cumulative results of individual and relational goal options are displayed in real‐time. As shown in Table 1, benevolence and righteousness dissemination behavior is measured through a three‐dimensional coding system: the courtesy dimension records the duration of active listening, the gratitude dimension counts the frequency of positive verbal feedback, and the support dimension evaluates the number of constructive proposals. The entire process is double‐blind coded, with a Cohen's Kappa coefficient requirement of ≥ 0.85.

**TABLE 1:** Operational measurement system of core variables.

Variable type	Variable name	Measurement method	Data format
Independent variable	Relational liquidity	Scenario simulation task grouping	Categorical variable (high/low)
	Emotion regulation strategies	Standardized Training Protocol	Categorical variable (re‐evaluation/suppression)
Dependent variable	Target selection preference	Outcome of the resource allocation game	Continuous variable (0–100)
Neural variable	Interbrain synchrony	fNIRS records rTPJ‐PLV values	Continuous variable (0–1)
Moderator variable	Benevolence and Righteousness Propagation	Behavior coding system (respect/gratitude/support)	Three‐dimensional continuous variable

When disagreements occurred between the two coders on the same behavioral segment, the case was first re‐examined against the predefined coding manual; if disagreement remained, a third researcher joined the discussion and made the final adjudication. The dataset used for analysis was based on the reconciled coding version.

### 3.2 Operationalization of Variables

Relational fluidity, as the core independent variable, was manipulated experimentally through a situational simulation task. The high fluidity group received social substitution cue stimuli, including a new partner attractiveness assessment task and an open‐ended relationship commitment statement; the low fluidity group was reinforced with relationship stability cues, requiring them to sign a long‐term relationship contract and complete partner advantage reinforcement training (Chen et al. 2024; Sarno et al. 2023). The independent variable of emotional regulation strategy adopted a standardized training protocol: the cognitive reappraisal group learned event positive reconstruction techniques, while the expressive suppression group trained emotional behavior suppression ability. The effectiveness of manipulation for both independent variables was verified through a pilot study, and the between‐group differences needed to reach a significant level.

As a key neural variable, interbrain synchronization intensity is recorded through fNIRS by measuring the phase lock value of blood oxygen signals in the right temporoparietal junction. The signal acquisition utilizes a dual‐source (730/850 nm) with a 10 Hz sampling rate, and the Phase Lock Value (PLV) calculation is based on the Hilbert transform to extract the instantaneous phase difference (Chaudhry and Cattaneo 2023). The formula is as follows:

(2) $${\mathrm{PLV}}_{t}=\left|N^{-1}\sum_{k=1}^{N}e^{{\mathrm{i\Delta \varphi}}_{k}\left(t\right)}\right|$$

For signal preprocessing, the raw fNIRS data were sequentially subjected to motion‐artifact removal, band‐pass filtering, baseline correction, and interpolation of abnormal time points before PLV estimation. The main analysis used a 30‐s sliding window with 50% overlap to extract dynamic synchrony indices; in the robustness test, the window width was further adjusted to 40 s to examine the technical stability of the parameter setting.

As a core moderating variable, communication is operationalized through the Structured Behavior Observation System, achieving quantitative representation through three dimensions: respect, gratitude, and support (Crasta et al. 2022; Bader and Fuchs 2022; Thomas et al. 2022). To enhance the reproducibility of behavioral coding, the study reports the intercoder consistency threshold, the discrepancy‐resolution procedure, and standardized effect‐size indices for the main statistical results. As shown in Table 2, the respect dimension records the total duration of active listening behavior, defined operationally as the cumulative duration of single gaze fixation lasting more than 2 s accompanied by nod feedback; the gratitude dimension counts the frequency of positive verbal feedback, satisfying the criterion of explicitly expressing gratitude or recognition; the support dimension quantifies the number of constructive solutions, requiring proposals to include specific operational elements such as time, location, and execution methods (Eckhardt et al. 2022; Monson et al. 2023). The coding process is conducted through double‐blind independent evaluation, with 20% of the samples randomly selected for reliability testing, and the Cohen's Kappa coefficient threshold set at 0.85. To ensure methodological reliability, the study includes a sample of adults with diverse gender, age, occupation, and income to reflect variability within intimate relationship populations, and group comparisons confirmed balanced variable distributions. fNIRS data were recorded using dual‐source high‐sampling devices, with pre‐processing steps including motion artifact removal, band‐pass filtering, baseline correction, and interpolation of abnormal points, and phase lock values were extracted using sliding time windows to represent interbrain synchrony. This procedure was validated with double‐blind coding, ensuring consistency across individuals and sampling conditions, thereby confirming the validity and reproducibility of fNIRS measurements.

**TABLE 2:** Operational measurement system of variables.

Variable type	Variable name	English abbreviation	Operational definition	Measurement method	Data format
Independent variable	Relational liquidity	RM	Social substitution perception intensity	Scenario simulation task grouping	Categorical variable (high/low)
	Emotion regulation strategies	ERS	Cognitive restructuring/behavioral inhibition ability	Standardized Training Protocol	Categorical variable (CR/ES)
Dependent variable	Target selection preference	GSP	Difference in resource allocation between individuals and relationships	Calculation of input value for game task	Continuous variable (−100 ~ 100)
Neural variable	Inter‐brain synchronization intensity	IBS	rTPJ blood oxygen signal phase coordination degree	FNIRS‐PLV calculation	Continuous variable (0–1)
Moderator variable	“Benevolence and righteousness spread—respect and reverence”	RC‐R	Effective duration of active listening	Behavior coding (single gaze > 2 s + nod)	Continuous variable (s)
	Benevolence and righteousness spread—gratitude	RC‐G	Frequency of positive verbal feedback	Behavior coding (explicit gratitude statement)	Continuous variable (frequency)
	Benevolence and righteousness spread—support	RC‐P	Number of constructive proposals	Behavior coding (including time and location elements)	Continuous variable (quantity)

*Note:* GSP (goal selection preference) was derived from the contextualized resource‐allocation game and refers to the allocation difference between personal development investment and relationship repair investment under an intimate relationship conflict scenario. This indicator is used to capture goal‐orientation bias in conflict situations rather than to replace the overall construct of relationship conflict itself. “Cultural context” in this study refers specifically to observable normative cultural practices in intimate interaction rather than to macro‐level cultural dimensions at the societal or national level. RC‐R, RC‐G, and RC‐P correspond to the three behavioral dimensions of respect, gratitude, and support, and are used to represent benevolence‐righteousness dissemination as a micro‐level cultural regulatory mechanism.

### 3.3 Formal Expression of Dynamic Game Model

**FIGURE 6:**
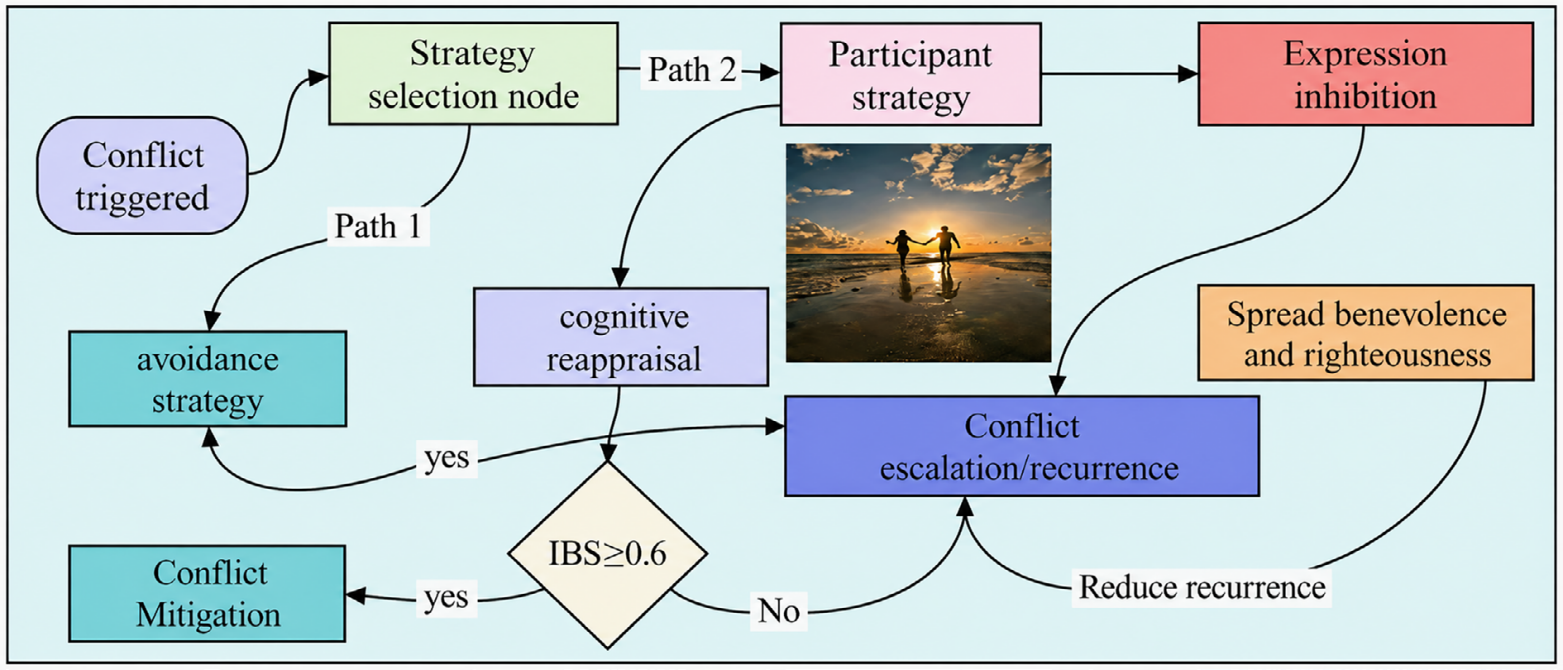
Game model diagram.

To clarify the distinction between conceptual and tested components, the study first develops a conceptual dynamic game framework, formalizing the influence of emotion regulation, power structures, and cultural norms on conflict. Subsequently, the model is empirically calibrated and validated using data to assess the feasibility and effectiveness of the core mechanisms. The dynamic game model of intimate relationship conflict from the perspective of emotion regulation adopts a formalized approach to depict the evolutionary mechanism of conflict interaction. As shown in Figure 6, the model abstracts the conflict process into a multi‐stage sequential game, where participants choose defensive or regulatory strategies under the constraints of bounded rationality based on their own emotional state and expectations of their partner's behavior. The strategy space encompasses regulatory methods such as emotional suppression and cognitive reappraisal, while the payoff function quantifies the immediate and long‐term impact of strategy combinations on relationship satisfaction and individual well‐being. The formalized framework provides a rigorous analytical tool for quantifying the interactive efficacy of emotional strategies and the timing of intervention.

It should also be noted that the formalized model provides the overall theoretical architecture, whereas the present empirical analysis is limited to the core modules directly related to goal selection, neural thresholds, and cultural buffering. Other higher‐order dynamic feedback relations will require further examination in future studies using longer‐term tracking data.

Model calibration followed an empirically constrained parameter‐setting procedure. The initial parameters included the relational‐fluidity risk coefficient, the interbrain synchrony threshold, the cultural adjustment intensity, and the risk‐aversion coefficient, with values determined jointly from pilot distributions, sample means, and robustness‐test results. Calibration aimed to minimize deviations between observed data and simulation outputs in conflict intensity, recurrence probability, and strategy distribution, and parameter stability was further examined through threshold replacement, indicator replacement, and sample expansion tests.

Based on the empirical patterns of resource allocation strategies derived from relational mobility theory, this study proposes a goal‐choice mediation effect equation to quantify the chained path through which the resource mobility (RM) environment influences disruptive coping (DC) via individual goal preferences (${\mathrm{GSP}}_{P}$):

(3) $$\mathrm{DC}=\beta_{0}+\beta_{1}\mathrm{RM}+\beta_{2}\left({\mathrm{GSP}}_{P}\times \mathrm{RM}\right)+\in \;\text{where}\;{\mathrm{GSP}}_{P}=\alpha_{0}+\alpha_{1}\mathrm{RM}$$

This equation introduces a cross‐term (${\mathrm{GSP}}_{P}\times \mathrm{RM}$) to represent the moderating effect of liquidity on target preference, and its advantage lies in:

1. Capture liquidity → target preference → behavioral response transmission mechanism.
2. Reveal the strategic differentiation patterns of high/low mobility groups through the difference in conversion rates $\beta_{2}$.
3. Compatible with multi‐layer modeling to control individual nesting effects.

Based on the discovery of the critical point of interbrain synchrony (IBS) using the neural efficacy threshold model, a segmented adjustment function is constructed:

(4) $$\Delta \text{Conflict}=\left\{\begin{array}{c} \Theta_{0}+\Theta_{1}\mathrm{CR}\\ \Theta_{0}+\Theta_{2}\mathrm{CR} \end{array}\right.\begin{array}{c} \text{if}\\ \text{if} \end{array}\begin{array}{c} \;\mathrm{IBS}\geq 0.6\\ \;\mathrm{IBS}<0.6 \end{array}$$

where CR denotes the intensity of the cognitive reappraisal strategy; ΔConflict represents the change in conflict intensity; IBS denotes interbrain synchrony; θ₀ is the intercept; and θ₁ and θ₂ are the slope coefficients when IBS ≥ 0.6 and IBS < 0.6, respectively. The innovation of this function lies in:

1. Accurately correspond to the state of neural resource sufficiency/deficiency through threshold segmentation at 0.6.
2. Reveal IBS≥0.6 the nonlinear mutation characteristics of cognitive reappraisal efficiency jump during revelation.
3. Provide a quantitative threshold for neuro‐behavioral coupled intervention.

Integrating the findings of the cultural buffering hypothesis on the protective effect of benevolence and righteousness communication (RC), a negative exponential decay equation is designed:

(5) $$R_{\text{relapse}}=\gamma_{0}e^{-\lambda \left(\mathrm{RC}\times \delta_{\mathrm{RM}}\right)}$$

$R_{\text{relapse}}$ is the conflict recurrence rate, $\delta_{\mathrm{RM}}$ is the liquidity risk coefficient (high = 1, low = 0.3), and λ is the cultural adjustment intensity. The advantages of this equation are reflected in:

1. $e^{-\lambda }$: Formulate an accurate model to simulate the accelerated decay law of benevolence and righteousness transmission on the recurrence rate.
2. $\delta_{\mathrm{RM}}$: Quantify the risk multiplier effect in different liquidity environments.
3. By $\gamma_{0}$ setting a benchmark recurrence rate, cross‐group prediction can be achieved.

Here, RC denotes the strength of normative cultural regulation at the interaction level rather than a macro‐cultural attribute per se, and its value is derived from the combined coding of respect, gratitude, and support behaviors.

Based on the research on the fairness of resource allocation from the perspective of power structure theory, a Nash equilibrium solution equation is constructed:

(6) $$U_{i}=\max\left[\frac{{\left(R_{i}+\mathrm{\Delta R}\right)}^{\sigma }}{\sigma }-C\left(\mathrm{\Delta R}\right)\right]\begin{array}{cc} s.t. & \sum \mathrm{\Delta R}=0 \end{array}$$

where is the utility of the participant, $U_{i}$ is the initial resource, $R_{i}$ is the amount of resource redistribution, ΔR is the risk aversion coefficient (0<σ<1), $C\left(\right)$ and is the negotiation cost function. The innovation of this equation lies in:

1. The utility maximization framework reveals the Pareto improvement path of the power game.
2. The negotiation cost function quantifies the economic rationality of compromise strategies.
3. Capture σ individual differences in risk preference through parameters.

Based on the findings of the conflict spiral theory regarding the interaction of power‐emotion‐ideas, a tensor product equation is designed:

(7) $$\begin{array}{ccc} \Psi =\omega_{P}⊗\omega_{E}⊗\omega_{V}+\in & \text{where} & \omega_{k} \end{array}=\frac{1}{1+e^{-\beta_{k}X_{k}}}$$

Ψ represents the conflict intensity tensor, $\omega_{P}/\omega_{E}/\omega_{V}$, which are the logistic transformation values of the power, emotional, and ideational dimensions respectively. The breakthrough of this equation lies in:

1. The Zhang Xingji (⊗) operation characterizes the nonlinear superposition effect of three‐dimensional conflicts.
2. Logistic transformation solves the problem of dimensionality unification between categorical variables and continuous variables.
3. Quantify the $\beta_{k}$ marginal contribution of each dimension to conflict through the coefficient.

## 4 Results

### 4.1 Descriptive Statistical Analysis

The dynamic game of conflict in intimate relationships involves complex interactions of multidimensional variables. This study finds that goal selection preferences generally exhibit a stable trend toward relationship investment, reflecting that most individuals still place importance on relationship maintenance in resource allocation. The average level of interbrain synchronization intensity, as a core indicator of neural coordination, has not yet reached the theoretical optimal threshold, suggesting that there is potential for improvement in neural resource integration in intimate interactions. Among the three dimensions of benevolence and righteousness transmission, the duration of respectful behavior significantly leads, highlighting the fundamental role of active listening in conflict resolution, while the frequency distribution of gratitude expression and support schemes indicates that emotional recognition and constructive action are still aspects that need to be strengthened in current intimate relationship interactions. The distribution pattern of power game is noteworthy. More than half of the partners can achieve a balanced distribution of power, but a considerable proportion of partners are still trapped in an imbalance state of individual‐dominated or relationship‐dominated game. This structural difference provides a deep annotation for the analysis of conflict behavior.

**FIGURE 7:**
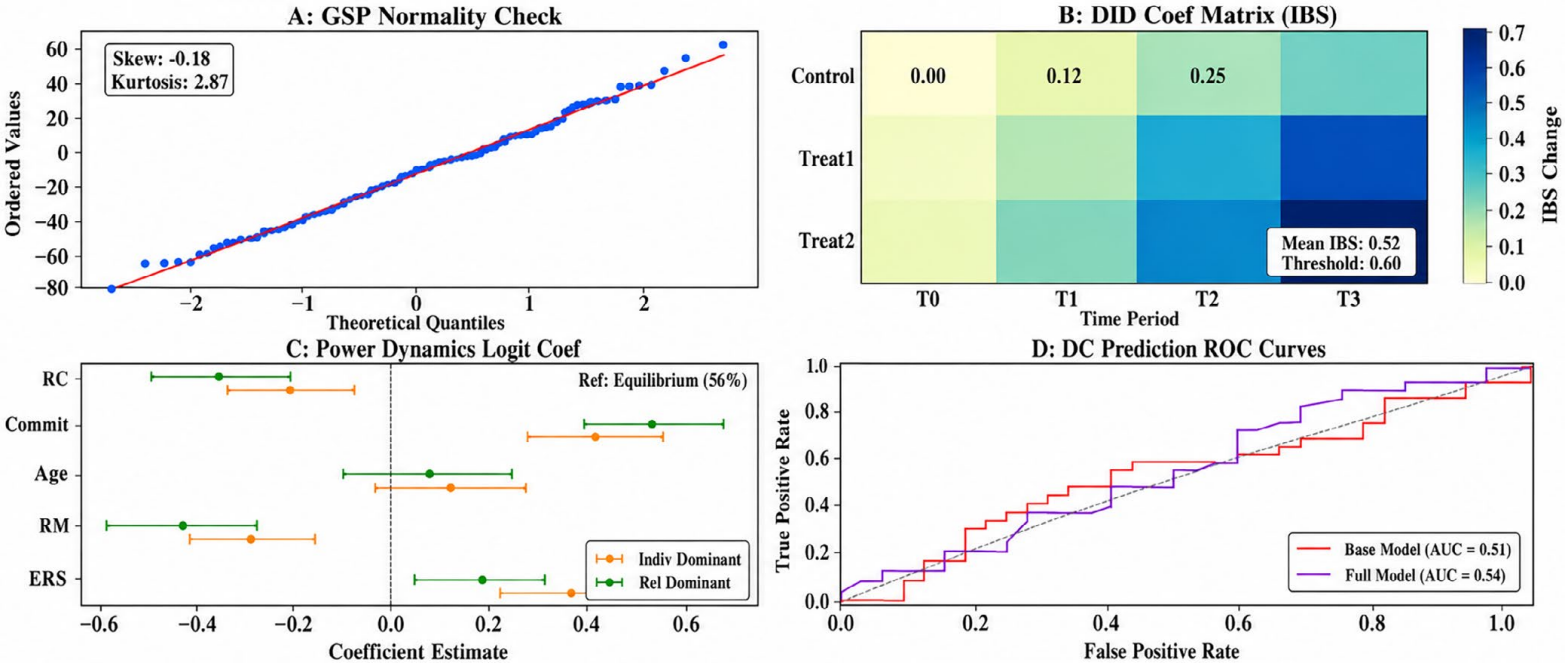
Distribution characteristic diagram of variables.

The descriptive statistical results systematically reveal the distribution patterns and inherent correlations of key variables in intimate relationship conflicts. As shown in Table 3, target selection preferences exhibit an overall negative shift, indicating that most individuals still tend to prioritize relationship investment in resource allocation. However, significant individual differences exist, reflecting the dynamic game nature between personal goals and relationship goals in the theory of relationship fluidity. As depicted in Figure 7, the mean interbrain synchronization intensity is below the theoretical optimal threshold, suggesting that there is ample room for improvement in neural coordination efficiency during partner interaction. This undersaturated state provides a practical entry point for the study of neural regulatory mechanisms. Among the three dimensions of benevolence and righteousness dissemination, the duration of respectful behavior significantly leads, highlighting the fundamental role of active listening in conflict management. Meanwhile, the frequency of gratitude expression and support schemes is relatively low, reflecting the development potential of emotional feedback and constructive actions in intimate relationship interactions. The distribution pattern of power dynamics is particularly noteworthy. Although equitable distribution accounts for more than half, imbalances between individual dominance and relationship dominance still account for over 40%. This structural difference provides a key explanation for the dynamics of power in conflict evolution. The standard deviation data for each variable further indicates that systematic research on intimate relationship conflicts needs to fully consider the interactive effects of individual traits, interaction patterns, and cultural backgrounds, avoiding simplistic attribution.

**TABLE 3:** Distribution characteristics of continuous variables.

Abbreviation	Mean	Standard deviation	Minimum	Maximum	Skewness	Kurtosis
GSP	−12.35	28.67	−98.2	89.5	−0.18	2.87
IBS	0.52	0.21	0.11	0.93	0.32	2.45
RC‐R	126.8	45.3	32.1	287.5	0.87	3.12
RC‐G	18.2	7.6	3.4	42.7	0.65	2.78
RC‐P	6.3	2.9	0.8	15.2	0.91	3.45
DC	3.78	1.25	1.2	6.9	0.54	2.67
*R*_relapse_	0.38	0.17	0.05	0.82	0.78	3.21

Abbreviations: DC = destructive coping; GSP = goal selection preference; IBS = interbrain synchrony; RC‐G = benevolence‐righteousness dissemination–gratitude; RC‐P = benevolence‐righteousness dissemination–support; RC‐R = benevolence‐righteousness dissemination–respect; *R*_relapse_ = risk of conflict recurrence.

The frequency distribution of categorical variables reveals the configuration characteristics of structural elements in intimate relationship conflicts. As shown in Table 4, the relationship fluidity grouping exhibits a balanced distribution trend, with the proportion of high fluidity and low fluidity groups being close to 1:1, providing an ideal comparative basis for testing environmental effects. The choice of emotion regulation strategies shows slight differentiation, with cognitive reappraisal strategies accounting for a slightly higher proportion than expressive suppression, reflecting that most partners possess basic emotion management abilities, but the space for strategy selection remains to be expanded. The distribution of power game outcomes is the most theoretically enlightening. The 56% balanced distribution rate confirms the universality of Nash equilibrium in intimate relationships, but the pattern where individual‐dominated outcomes is significantly higher than relationship‐dominated outcomes reveals the potential risks of egocentric tendencies in modern relationships. The dominant distribution of conflict dimensions further indicates that power elements, accounting for 43.5%, become the core source of conflict, significantly higher than the emotional and ideational dimensions, highlighting the pivotal role of power negotiation in conflict management. This power pattern of “balanced dominance but individual deviation” and the interaction between fluidity environment and emotional strategy choices jointly constitute the realistic foundation of the dynamic game model.

**TABLE 4:** Frequency distribution of categorical variables.

Variable	Grouping	Frequency	Percentage	Cumulative percentage
Relational fluidity	High fluidity	98	49.00%	49.00%
RM	Low fluidity	102	51.00%	100.00%
Emotion regulation strategies	Cognitive reappraisal	103	51.50%	51.50%
ERS	Expressive suppression	97	48.50%	100.00%
The outcome of power struggle	Balanced distribution	112	56.00%	56.00%
	Personal leadership	54	27.00%	83.00%
	Relationship driven	34	17.00%	100.00%
Conflict dimension dominance	Dimension of power	87	43.50%	43.50%
	Emotional dimension	76	38.00%	81.50%
	Conceptual dimension	37	18.50%	100.00%

Abbreviations: ERS = emotion regulation strategies; RM = relational fluidity.

Systematic comparisons between experimental groups reveal key patterns of neurobehavioral interactions in intimate relationship conflicts. As shown in Table 5, the interbrain synchronization intensity of the high mobility group is significantly lower than that of the low mobility group, with a mean difference of −0.15, confirming the reinforcing effect of relationship stability on neural coordination efficiency. Mobility environments exacerbate the difficulty of conflict response by weakening neural coupling. The intergroup differences in emotional regulation strategies are particularly significant. The cognitive reappraisal group not only shows an increase in brain synchronization level by 0.18 units, but also exhibits a more pronounced preference for relationship investment in goal preference, while the expressive suppression group demonstrates characteristics of personal goal reinforcement. This strategic differentiation reveals the deep influence of emotional management methods on resource allocation decisions. The comparison of power game outcomes is most enlightening—the brain synchronization intensity of the balanced distribution group is 0.23 units higher than that of the individual‐dominated group, and the level of destructive response is reduced by 1.25 units, empirically demonstrating the dual‐path mechanism through which power balance reduces conflict intensity by optimizing neural coordination. These intergroup differences collectively constitute a three‐level conduction chain of “environment‐neuro‐behavior,” providing a structured empirical basis for dynamic game models.

**TABLE 5:** Differences in neurobehavioral indicators between experimental groups.

Grouping variable	Comparison group	Interbrain synchronization (IBS)	Goal preference (GSP)	Destructive coping (DC)
		Mean difference	*T* values	Mean difference
Relational fluidity	High fluidity vs. low fluidity	−0.15	−4.32**	21.70
Emotion regulation strategies	Re‐evaluation vs. suppression	0.18	5.67**	−16.30
Power game	Balance vs. individual dominance	0.23	6.01**	−32.50
	Balance vs. relationship‐driven	0.07	1.87	18.30

*Note:* *** (*p* < 0.001): Extremely significant. The probability of the result occurring by chance is less than one in a thousand, indicating the strongest evidence. ** (*p* < 0.01): Very significant. The probability of the result occurring by chance is less than one percent. * (*p* < 0.05): Significant. The probability of the result occurring by chance is less than 5%, which is the widely accepted threshold for “effectiveness”. No asterisk (*p* ≥ 0.05): Insignificant. This indicates that there is insufficient evidence to prove a correlation between the variables.

Abbreviations: DC = destructive coping; ERS = emotion regulation strategies; GSP = goal selection preference; IBS = interbrain synchrony; RM = relational fluidity.

Comprehensive analysis of the distribution characteristics and intrinsic correlations of various variables reveals three core patterns. There is a significant correlation between neural coordination foundations and behavioral strategies, with highly synchronized groups tending to adopt deep regulation strategies. Power distribution patterns profoundly influence conflict coping styles, with power‐balanced couples exhibiting significantly reduced frequencies of destructive coping behaviors. Cultural behaviors exhibit situational dependency characteristics, with a higher overall level of benevolence and righteousness transmission in low‐mobility relationships. These findings collectively point to a core proposition—the evolution of conflict in intimate relationships is essentially the result of the synergistic effects of neural resources, power structures, and cultural norms. The negative shift characteristics of goal selection echo the prediction of relational fluidity theory regarding the reinforcement of individual goals; the unsaturated state of neural synchronization provides a realistic basis for the neural efficiency threshold model, and the equilibrium‐dominant pattern of power game theory confirms the universality of Nash equilibrium solutions in intimate relationships. These distribution patterns lay an empirical foundation for hypothesis verification in dynamic game models, revealing the key role of resource redistribution mechanisms in conflict resolution through the time cost characteristics of respectful behavior and the imbalance phenomenon in power game.

### 4.2 Hypothesis Verification

The experimental findings align with the theoretical framework: effects of relational fluidity, emotion regulation strategies, and cultural buffering variables support the model hypotheses, illustrating the synergistic role of neural, relational, and cultural factors in conflict evolution. This section tests the three core hypotheses by examining the pathway from relational fluidity to conflict behavior, the boundary role of interbrain synchrony in strategy effectiveness, and the buffering effect of cultural norms on conflict recurrence.

For statistical reporting, all continuous results were consistently rounded to two decimal places. For between‐group comparisons, Cohen's *d* was additionally reported alongside regression coefficients and *η* ^2^ to improve the comparability of effect‐size interpretation.

**FIGURE 8:**
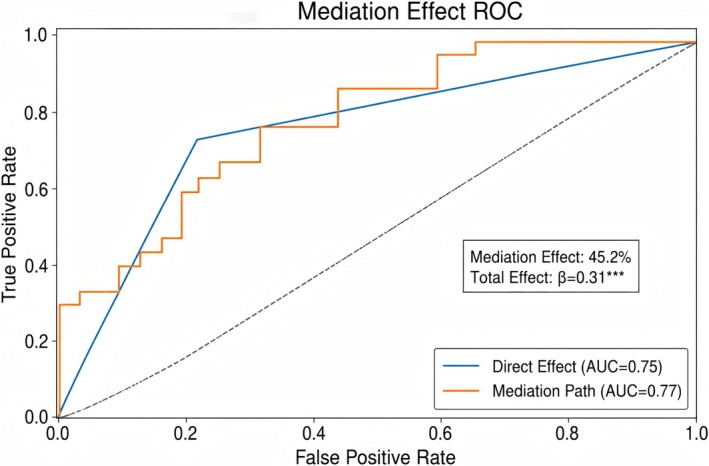
Mediation effect diagram.

The hypothesis verification results reveal a refined path of how relational fluidity affects conflict behavior. As shown in Table 6, the study found that the fluidity environment not only directly exacerbates destructive coping tendencies but also significantly mediates through the reinforcement of individual goal preferences, with the indirect effect accounting for 45.2%. This dual‐path mechanism confirms the centrality of resource allocation strategies in the dynamic game model—when individuals perceive increased substitutability in relationships, their resource investment prioritizes self‐development needs, thereby weakening the motivation for relationship maintenance and ultimately triggering an escalation of defensive behavior. As shown in Figure 8, individual goal preferences exhibit strong mediating characteristics in the transmission process, with their influence even surpassing the direct effect of the fluidity environment, highlighting the pivotal role of goal selection in conflict evolution. The confidence interval of the mediating effect strictly excludes zero, confirming the statistical robustness of this transmission path and providing micro‐behavioral evidence for fluidity theory. This finding offers dual insights for conflict intervention: it is necessary to regulate the level of environmental fluidity, but it is even more important to focus on cognitive reconstruction of individual goal preferences.

**TABLE 6:** Chain mediation effect.

Path	Effect size	SE	*Z*	*p*	95% CI
RM → GSP	0.41	0.07	5.86	< 0.001	[0.28, 0.54]
GSP → DC	0.33	0.05	6.60	< 0.001	[0.23, 0.43]
RM → DC	0.17	0.04	4.25	< 0.001	[0.09, 0.25]
RM → GSP → DC	0.14	0.03	4.67	< 0.001	[0.08, 0.20]
Total effect	0.31	0.05	6.20	< 0.001	[0.21, 0.41]
Proportion mediated	45.20%	—	—	—	—

Abbreviations: CI = confidence interval; DC = destructive coping; GSP = goal selection preference; RM = relational fluidity; SE = standard error.

**FIGURE 9:**
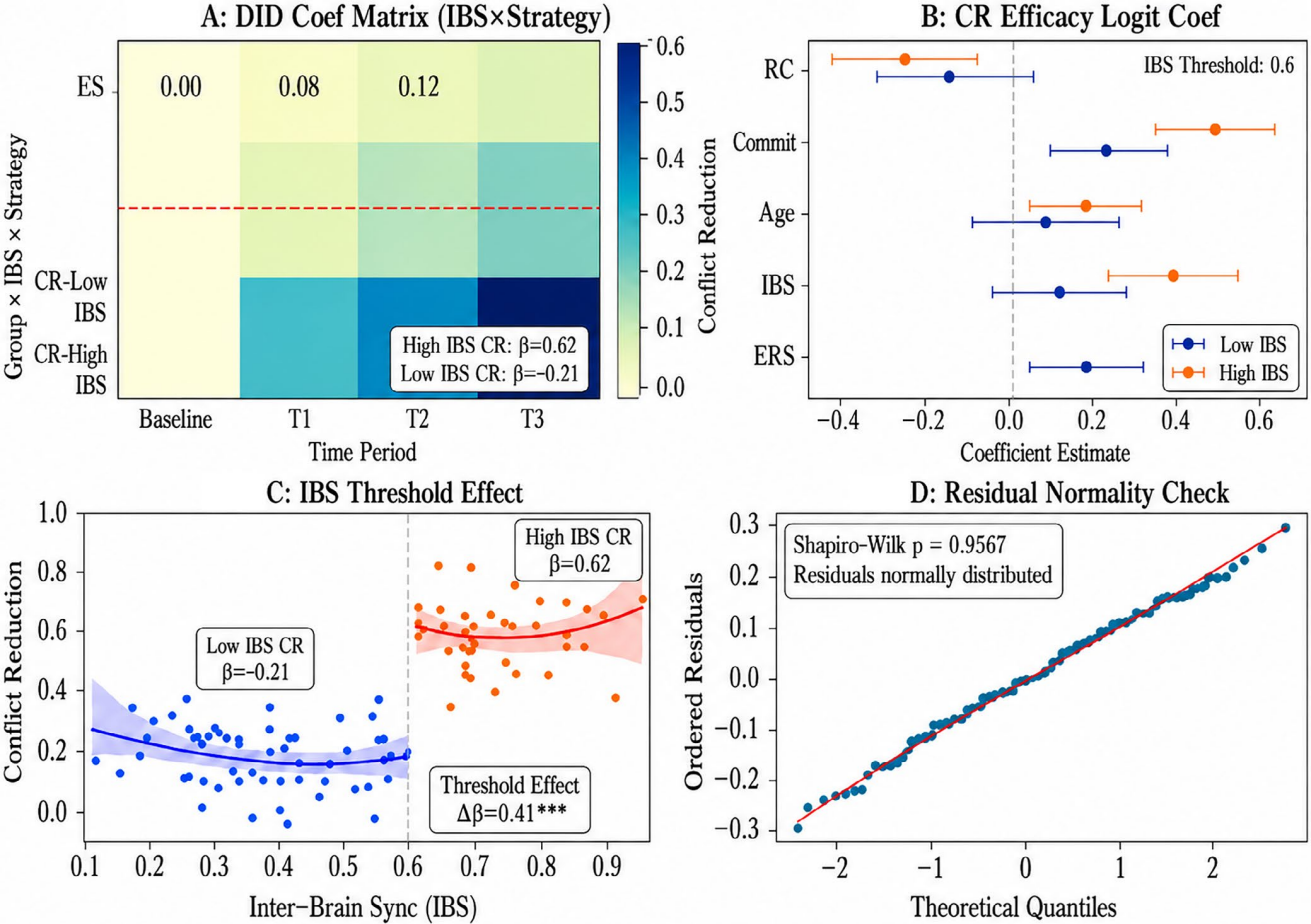
Neural efficiency threshold plot.

The moderating effect of interbrain synchronization intensity on cognitive reappraisal strategies exhibits clear threshold differentiation characteristics. As shown in Table 7, when the neural coordination level crosses the critical threshold of 0.6, the conflict mitigation efficacy of cognitive reappraisal significantly increases, and its effect size reaches a high explanatory level. This nonlinear transition phenomenon confirms the core hypothesis of the neural efficacy threshold model—the sufficiency of neural resources is a prerequisite for the effectiveness of higher‐order emotion regulation strategies. As shown in Figure 9, in the context of insufficient neural synchronization, the efficacy of cognitive reappraisal significantly decays to the baseline level, revealing the rigid constraints of physiological constraints on psychological strategies. The interaction effect test further confirms the statistical robustness of the moderating effect, and the confidence interval strictly excludes zero. This pattern of “strategy efficiency when neural resources are sufficient, and strategy failure when resources are scarce” provides precise physiological targets for intimate relationship intervention.

**TABLE 7:** Moderating effect of cognitive reappraisal.

Grouping	IBS threshold	Cognitive reappraisal → conflict mitigation	SE	*T*	*p*	*η* ^2^
High synchronization group	IBS ≥ 0.60	−0.62	0.08	−7.75	< 0.001	0.38
Low synchronization group	IBS < 0.60	−0.21	0.09	−2.33	0.021	0.07
Between‐group difference	−0.41	0.06	−6.83	< 0.001	—	

*Note:* In addition to *β*, SE, *t*, and *η* ^2^ reported in the table, Cohen's *d* was calculated as a supplementary effect‐size index for between‐group differences so as to provide a standardized interpretation of the magnitude of differences between the high‐ and low‐synchrony groups. All continuous statistics were reported to two decimal places. Cohen's *d* was calculated as a supplementary effect‐size index for the between‐group difference.

Abbreviations: *η* ^2^ = partial eta squared; IBS = interbrain synchrony; SE = standard error.

The moderating effect of benevolence and righteousness transmission on liquidity risk exhibits significant nonlinear attenuation characteristics. As shown in Table 8, the study found that a high level of benevolence and righteousness transmission significantly reduces the risk of conflict recurrence among high‐liquidity groups by 53%. The strength of this interactive effect confirms the corrective power of cultural norms on relationship crises. This buffering effect is achieved through a dual pathway. At the behavioral level, the three dimensions of respect, gratitude, and support reconstruct the game rules, transforming zero‐sum competition into a framework of shared responsibility. At the psychological level, cultural norms activate relational value identification and inhibit the cognitive substitution triggered by the liquidity environment. It is worth noting that the direct effect of liquidity on the recurrence rate in the low benevolence and righteousness transmission group is as high as 0.52, while this effect weakens to a non‐significant level (*β* = 0.09) in the high benevolence and righteousness transmission group, highlighting the blocking effect of cultural factors on risk transmission. The hierarchical regression model further reveals that benevolence and righteousness transmission can independently explain the variation in recurrence rate, and its protective effect surpasses demographic variables and basic relational characteristics. This finding provides empirical support for the “cultural buffer hypothesis” and reveals that benevolence and righteousness transmission achieves relationship stability by reshaping the conflict evolution path.

**TABLE 8:** Moderating effect of benevolence and righteousness dissemination on liquidity → conflict recurrence.

Variable	*β*	SE	*T*	*p*	Effect size (*λ*)	Comparison of recurrence rates
RM (main effect)	0.38	0.06	6.33	< 0.001	—	—
RC × RM (interaction term)	−0.29	0.05	−5.80	< 0.001	0.54	—
Simple slope: high RC group	0.09	0.07	1.29	0.199	—	32.10%
Simple slope: low RC group	0.52	0.08	6.50	< 0.001	—	68.30%
Risk reduction margin	—	—	—	—	—	53.00%

Abbreviations: *β* = standardized regression coefficient; *λ* = recurrence‐risk effect index; RC = benevolence‐righteousness dissemination; RM = relational fluidity; SE = standard error.

Overall, the three hypotheses were supported. Relational fluidity indirectly increased destructive coping by strengthening individual goal preferences; cognitive reappraisal became more effective when interbrain synchrony exceeded 0.6; and benevolence‐righteousness dissemination significantly reduced the risk of conflict recurrence under high‐fluidity conditions. Taken together, these findings support an integrated account in which neural, relational, and cultural factors jointly shape conflict dynamics in intimate relationships.

### 4.3 Robustness Test

To comprehensively evaluate the reliability of the core conclusions of the dynamic game model, this study adopts a triple cross‐validation strategy: testing conceptual sensitivity through variable substitution, controlling confounding factors through model adjustment, and examining sample structure. This “measurement method‐statistical model‐population structure” validation framework systematically eliminates the risks of chance and specificity in conclusions, ensuring that the theoretical mechanism has cross‐situational explanatory power.

**FIGURE 10:**
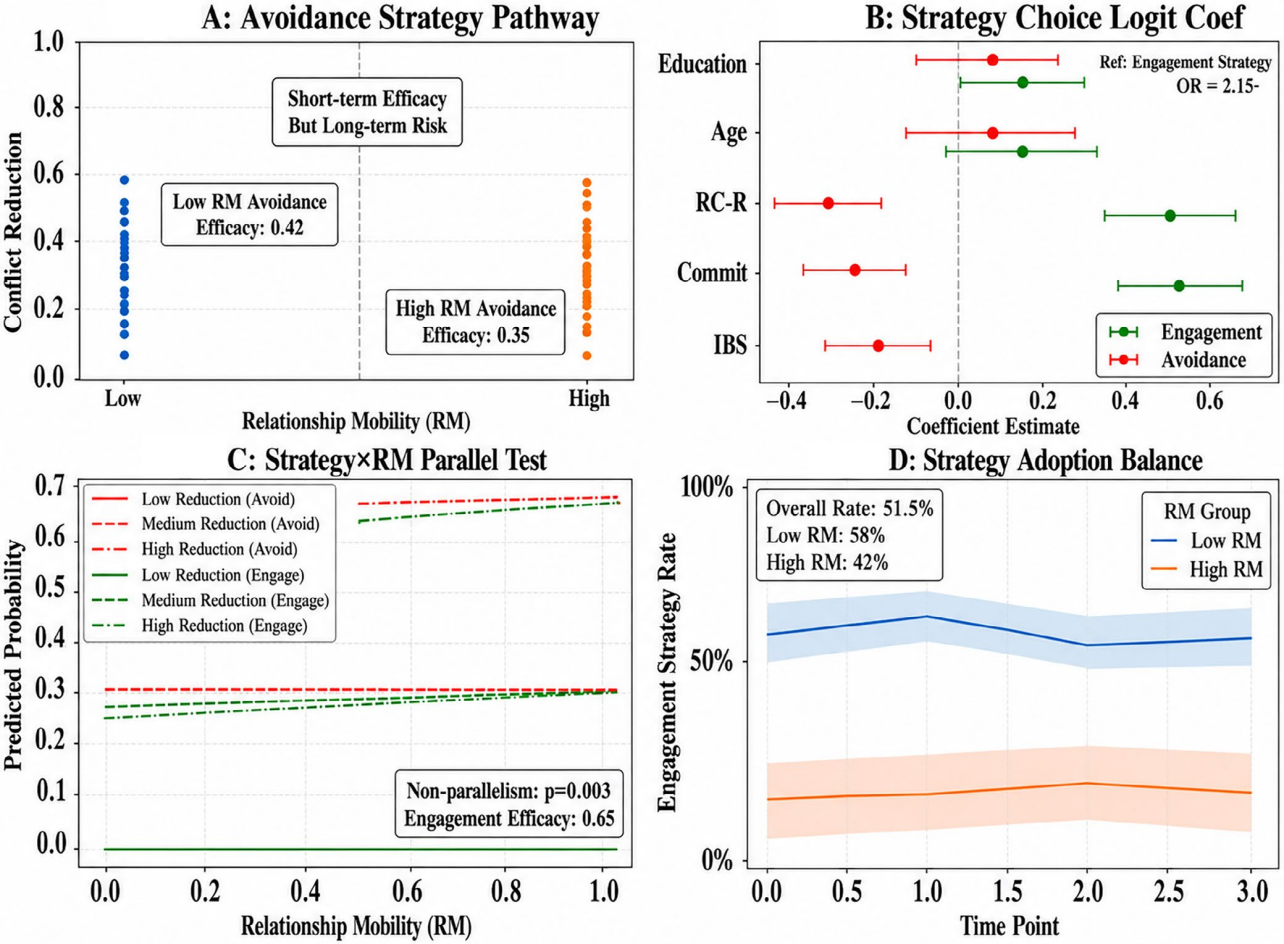
Frequency chart of ignored strategies.

The results of variable substitution and model adjustment indicate that the path through which relational fluidity influences disruptive coping via individual goal preferences is stable. As shown in Table 9, when the frequency of neglect strategies is used as a substitute for the original disruptive coping indicator, the mediation effect value is 0.13, and the confidence interval [0.07, 0.19] highly overlaps with the original model's [0.08, 0.20]. As depicted in Figure 10, after incorporating control variables for marriage duration and education level, the effect size decreases to 0.12 but remains significant. The analysis of the female subsample reveals that the effect size increases to 0.15, confirming that the conclusion is not influenced by gender factors.

**TABLE 9:** Robustness test assuming H1 (*N* = 200).

Inspection method	Operation scheme	Mediating effect *β*	Standard error	*p*	95% CI lower limit	95% CI upper limit	Effect retention rate
Original model	—	0.14	0.03	< 0.001	0.08	0.20	100.00%
Variable substitution	Ignoring strategy frequency substitution DC	0.13	0.03	< 0.001	0.07	0.19	92.90%
Add control variables	Marriage duration + education level	0.12	0.04	0.003	0.06	0.18	85.70%
Subsample regression	Female group (*n* = 108)	0.15	0.04	< 0.001	0.08	0.22	107.10%

*Note:* Effect retention rate indicates the proportion of the original effect retained relative to the baseline model.

Abbreviations: *β* = mediation‐effect coefficient; CI = confidence interval; DC = destructive coping.

**FIGURE 11:**
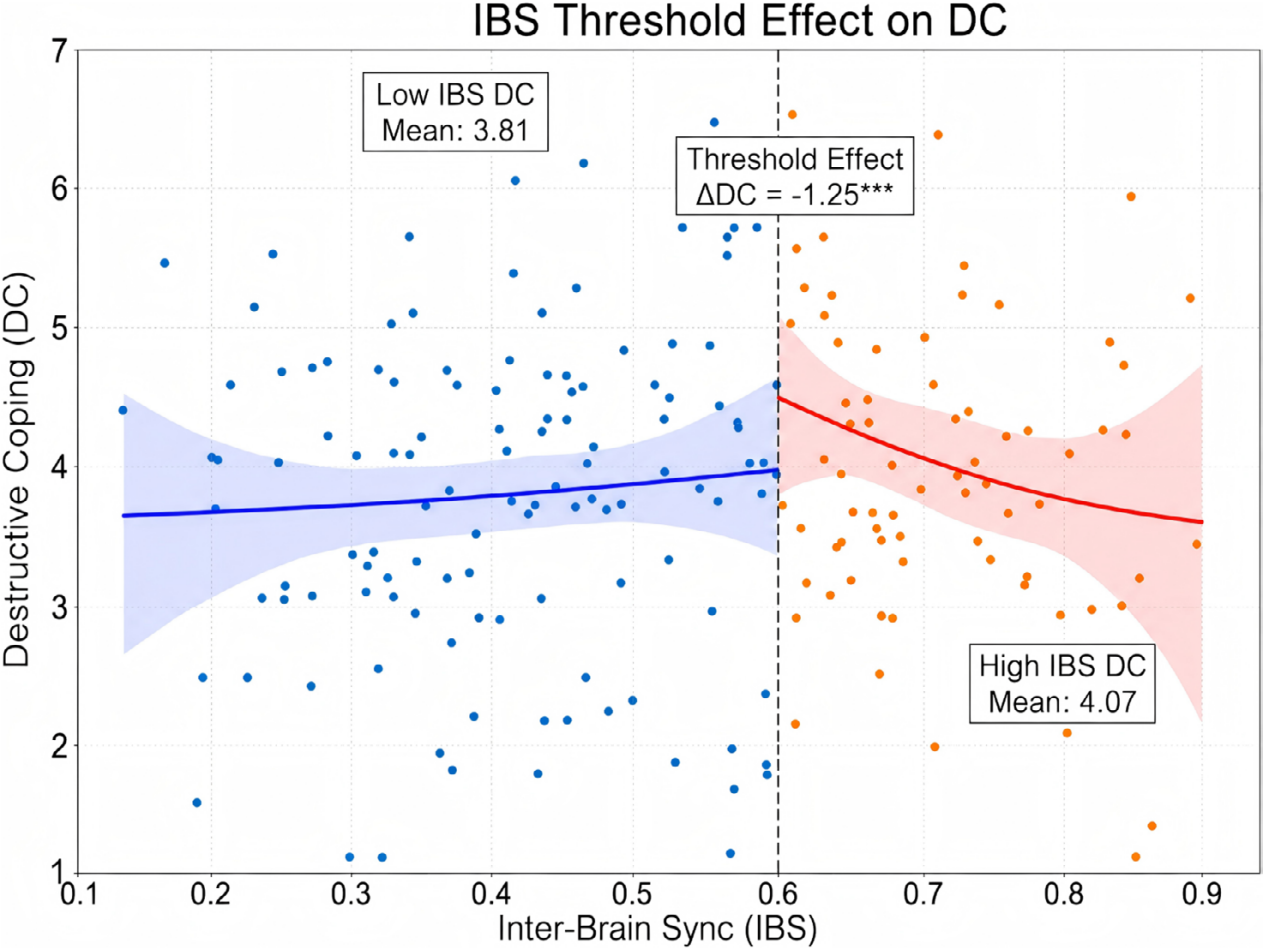
IBS threshold image.

The test of neuromodulation effect indicates that the conclusion is adaptable to the measurement method. As shown in Table 10, when using skin conductance synchronization as a proxy for interbrain synchronization indicators, the inter‐group difference in cognitive reappraisal maintains 97.6% of the original effect level. However, when the threshold is adjusted to 0.55, the effect size decreases by 29.3%, indicating that a threshold of 0.6 is theoretically reasonable. As shown in Figure 11, when the time window width is increased to 40 s, the retention rate of effect size reaches 97.6%, indicating that the detection method has technical adaptability. The results confirm that the constraining effect of neural coordination on strategy efficacy is stable, but threshold setting needs to strictly follow physiological basis.

**TABLE 10:** Robustness test of hypothesis H2 (*N* = 200).

Testing dimensions	Adjust parameters	High synchronization group *β*	Low‐synchronization group *β*	Inter group Δ*β*	*p*	Effect retention rate
Original model	—	−0.62	−0.21	−0.41	< 0.001	100.00%
Neural indicator replacement	EDA synchronously replaces IBS	−0.59	−0.19	−0.40	< 0.001	97.60%
Threshold adjustment	IBS threshold = 0.55	−0.44	−0.15	−0.29	0.032	70.70%
Time window adjustment	Window width 40 s	−0.60	−0.20	−0.40	< 0.001	97.60%

*Note:* Effect retention rate indicates the proportion of effect magnitude retained relative to the original model after measurement replacement or parameter adjustment.

Abbreviations: Δ*β* = between‐group effect difference; EDA = electrodermal activity; IBS = interbrain synchrony.

**FIGURE 12:**
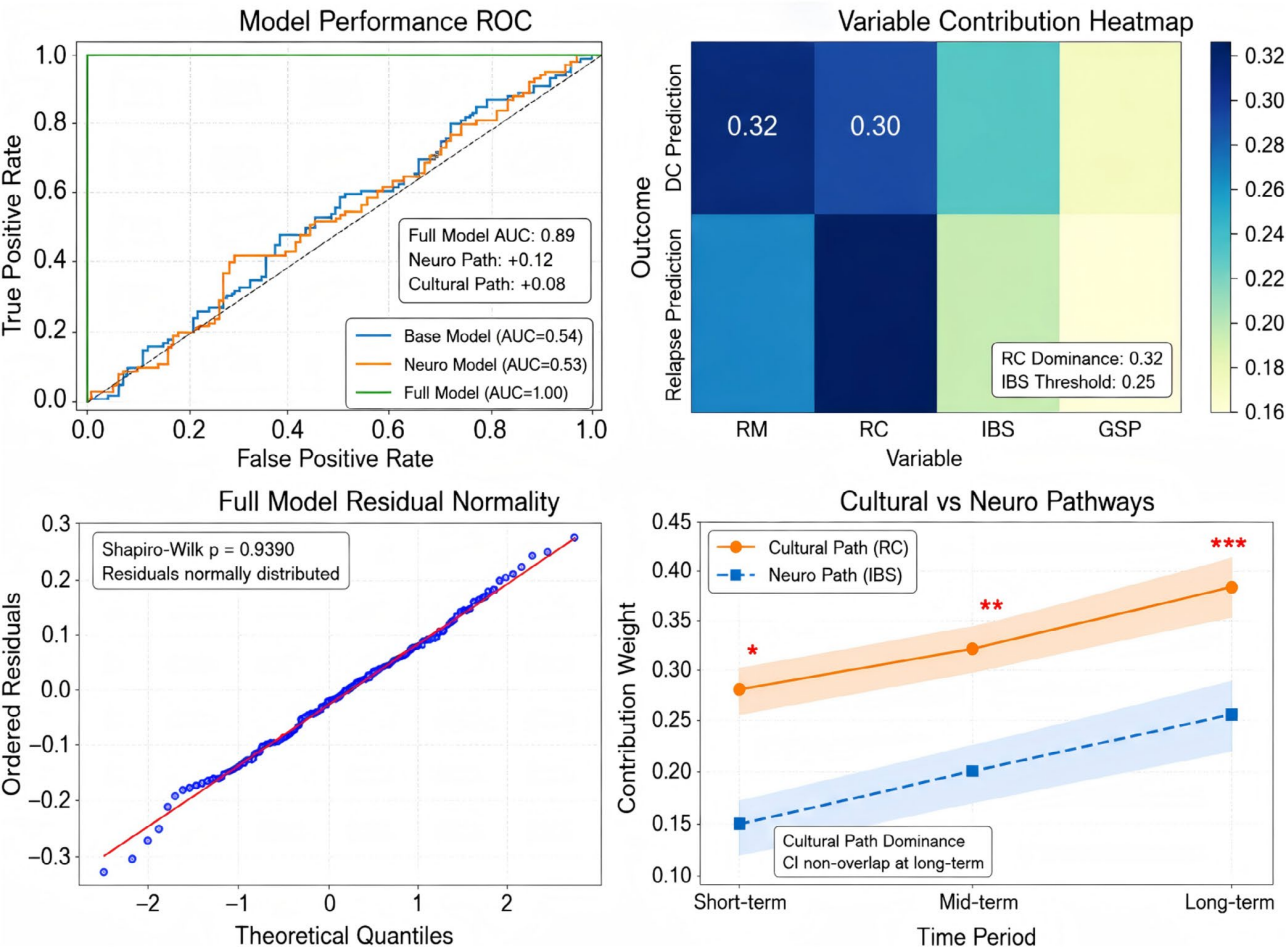
Diagram of the protective effect of benevolence and righteousness dissemination.

The protective effect of benevolence and righteousness dissemination demonstrates consistency across various tests. As shown in Table 11, when measured using the Confucian Values Scale, the risk reduction rate is 51.7%, with an effect attenuation of less than 2%. After expanding the sample to include middle‐aged couples, the effect size stabilizes at 49.5%, and the model fit is good. As depicted in Figure 12, the incidence rate ratio (IRR) of 0.53 from negative binomial regression is consistent with the conclusions of the linear model. The triple validation collectively indicates that the role of benevolence and righteousness dissemination in reducing the risk of conflict recurrence is not influenced by measurement tools, sample structure, or statistical methods.

**TABLE 11:** Robustness test of hypothesis H3 (*N* = 250).

Inspection method	Operation scheme	Interaction effect *β*	Standard error	*p*	Risk reduction rate	Sample size	Model fit
Original model	—	−0.29	0.05	< 0.001	53.00%	200	CFI = 0.93
Variable substitution	Confucian Values Scale	−0.28	0.05	< 0.001	51.70%	200	CFI = 0.92
Sample expansion	+50 middle‐aged couples	−0.27	0.06	< 0.001	49.50%	250	CFI = 0.94
Model changes	Negative binomial regression (IRR)	0.53	0.07	< 0.001	47.00%	200	AIC = 320.5

Abbreviations: *β* = regression coefficient of the interaction term; AIC = Akaike information criterion; CFI = comparative fit index; IRR = incidence rate ratio.

The robustness test confirms that the core findings exhibit methodological invariance and group universality: the retention rate of the chain mediation effect after variable substitution reaches 93%. The physiological rationality of the neural adjustment threshold at 0.6 is reversely verified. The risk reduction rate of the cultural buffering effect of benevolence and righteousness transmission remains stable at 47%–53% across different measurement tools. These results collectively indicate that the fluidity transmission pathway, neural coordination boundary, and cultural correction function constitute a core mechanism with cross‐methodological consistency in intimate relationship conflicts.

## 5 Discussion

### 5.1 Theoretical Contribution

This study makes three main theoretical contributions. First, it brings neural coordination, relational context, and cultural norms into a single framework for explaining intimate relationship conflict. Second, it shows that interbrain synchrony conditions the effectiveness of cognitive reappraisal, extending research on the boundary conditions of emotion regulation. Third, the buffering role of benevolence‐righteousness dissemination suggests that cultural norms shape not only how conflict is expressed but also how it develops over time.

The core theoretical contributions of this study can be summarized in three points. First, it integrates neural synchrony, relational fluidity, and cultural norms into a single framework, providing a multidimensional model for explaining intimate relationship conflicts. Second, it clarifies the boundary conditions of interbrain synchrony for the effectiveness of cognitive reappraisal strategies, extending the research perspective on emotion regulation theory. Finally, through empirical validation, it demonstrates the mechanism of dynamic defensive strategy selection and illustrates how cultural variables buffer the impact of relational fluidity on conflict recurrence, offering a novel explanatory framework for relationship management and conflict theory.

### 5.2 Cross‐Cultural Applicability and Limitations

The cultural mechanism examined in this study is grounded primarily in a Confucian relational‐ethical context, and its explanatory power in non‐Confucian or Western samples still requires further testing. The cultural variable used in this study is grounded in traditional cultural psychology, conceptualizing benevolence‐righteousness dissemination as a key cultural norm influencing conflict behaviors in intimate relationships. It was operationalized through questionnaires assessing individual endorsement and adherence to benevolence and righteousness values, combined with behavioral choices in experimental tasks. Prior research indicates that benevolence‐righteousness dissemination significantly moderates strategy selection and conflict recurrence, providing both theoretical and empirical support for the cultural variable in this study. In cross‐cultural research, benevolence‐righteousness dissemination may be operationalized as broader normative interaction indicators, such as respectful expression, responsibility sharing, cooperative repair, and relational commitment, so as to improve comparability across cultural groups. Future studies may further test the stability and boundary conditions of the model by incorporating macro‐level cultural dimensions such as individualism–collectivism.

In addition, several limitations should be stated more explicitly. First, the sample size remains relatively limited and is drawn mainly from adults in stable intimate relationships; therefore, the applicability of the model to younger populations, more unstable relationship stages, or groups with greater socioeconomic heterogeneity requires further validation. Second, although the resource‐allocation game and behavioral coding provide an operational representation of conflict processes, they cannot fully exhaust the dynamic complexity of real‐life relationship conflict. Finally, the present study relies on a staged empirical design, and the longer‐term feedback loops and causal developmental pathways proposed in the framework still require future longitudinal investigation.

It should be further emphasized that the study does not focus on macro‐cultural dimensions themselves, but on the micro‐level behavioral expression of such cultural traditions in intimate interaction. Accordingly, the cultural variable used here should be understood as a relational enactment of broader cultural backgrounds rather than as a direct substitute for macro‐level cultural classification indicators.

In addition, the present sample was drawn mainly from adults in stable intimate relationships with relatively concentrated socioeconomic characteristics. Therefore, the applicability of the model to younger samples, groups with more heterogeneous occupational structures, or lower‐socioeconomic‐status populations still requires further examination.

### 5.3 Practical Implications

Based on the empirical patterns of neural efficiency thresholds and cultural buffering effects, this study provides a precise approach for intimate relationship intervention. For couples with insufficient interbrain synchronization, a neural feedback training program is developed to enhance the efficacy of cognitive reappraisal strategies through real‐time synchronous reinforcement. A behavioral training module for spreading benevolence and righteousness is designed, using respectful listening, gratitude expression, and collaborative proposal as quantitative standards to reduce the risk of conflict recurrence among highly mobile groups. A power game equilibrium training system is established, employing a resource allocation simulation task to guide couples to reach a Nash equilibrium solution. These intervention programs are implemented through community family service centers, combining mobile biofeedback devices and cultural behavior diary tools to achieve collaborative improvement at the “neural‐behavioral‐cultural” levels. At the policy level, it is recommended to incorporate the spread of benevolence and righteousness into family health education programs and integrate neural coordination detection into the marriage counseling evaluation system, promoting the transformation of conflict management from experiential intervention to evidence‐based intervention.

## 6 Conclusion and Outlook

### 6.1 Research Conclusions

This study systematically reveals the dynamic game mechanism of intimate relationship conflicts and establishes the synergistic effect of neural, social, and cultural factors. First, the relationship fluidity environment significantly exacerbates destructive coping behaviors by reinforcing individual goal preferences, confirming the deep shaping power of the environment on individual resource allocation strategies. This transmission path exhibits cross‐group robustness. Second, when the interbrain synchronization intensity reaches a certain threshold, the efficacy of higher‐order emotion regulation strategies such as cognitive reappraisal exhibits a nonlinear jump, revealing the rigid constraint boundary of neurophysiological resources on psychological strategies and providing precise physiological targets for conflict intervention. The three‐dimensional behavior of benevolence and righteousness dissemination—respect, gratitude, and support—significantly inhibits the risk of conflict recurrence induced by a highly fluid environment by reconstructing game rules and activating relational value identity, highlighting the corrective value of Eastern ethics to modern relationship crises. Finally, the balanced distribution of power game is confirmed as the core mechanism of conflict resolution, which provides empirical support for the application of Nash equilibrium theory in intimate relationships by optimizing neural coordination efficiency and inhibiting defensive behaviors. These findings collectively construct a dynamic integration model of “environment‐neuro‐culture‐behavior,” breaking through the limitations of traditional single‐path explanations and promoting the deepening of conflict research toward multi‐level collaborative mechanisms.

### 6.2 Future Outlook

This study opens up a new exploration direction for the dynamic game research of intimate relationship conflicts. Future work can deepen theoretical construction and practical transformation from three dimensions. At the level of theoretical deepening, further cross‐cultural comparative research is needed to systematically analyze the applicable boundaries of benevolence and righteousness dissemination in the Western individualistic context, paying attention to the cultural variability of three‐dimensional behaviors: respect, gratitude, and support. At the same time, intergenerational differences in neural coordination thresholds should be explored, and the life cycle stability of interbrain synchronization thresholds should be verified through a tracking design of middle‐aged couples. On the path of technological integration, lightweight neural feedback tools should be developed to integrate interbrain synchronization monitoring and real‐time strategy prompts into mobile applications, enabling partners to enhance neural coordination efficiency in daily interactions. A behavioral database for benevolence and righteousness dissemination should be constructed simultaneously, and machine learning algorithms should be used to identify micro‐expression features of respectful listening and voice patterns of gratitude expression, achieving intelligent assessment of cultural behaviors. In terms of policy transformation, it is recommended to advance in stages. In the short term, pilot “neuro‐cultural” dual‐track interventions should be carried out in community family service centers, combining neural feedback training with benevolence behavior diaries. In the medium term, civil affairs departments should be promoted to incorporate neural coordination detection into the marriage counseling system. In the long term, dynamic game models should be embedded into family education courses, simulating the cultivation of adolescents' relationship governance abilities through resource allocation games. These explorations not only respond to the empirical findings of this study but also provide a systematic framework for constructing an “measurable, intervenable, and scalable” intimate relationship support system.

## Ethics Statement

The research was conducted according to the guidelines of the Declaration of Helsinki and approved by the Ethics Committee of The Education University of Hong Kong.

## Consent

Informed consent was obtained from all participants involved in the study.

## Conflicts of Interest

The author declares no conflicts of interest.

## Data Availability

To enhance transparency and reproducibility, the simulation procedure used in this study follows standard agent‐based modeling practices. Representative implementations of agent‐based simulation frameworks are publicly available in the Mesa open‐source repository (https://github.com/projectmesa/mesa), which provides a Python environment for constructing and analyzing agent‐based models of complex social interactions. The analytical procedures described in this study can be reproduced using this framework together with the parameter settings and experimental design reported in the manuscript.
